# Vitamin D deficiency is significantly associated with depression in patients with chronic kidney disease

**DOI:** 10.1371/journal.pone.0171009

**Published:** 2017-02-13

**Authors:** Jong Hyun Jhee, Hyoungnae Kim, Seohyun Park, Hae-Ryong Yun, Su-Young Jung, Youn Kyung Kee, Chang-Yun Yoon, Jung Tak Park, Seung Hyeok Han, Shin-Wook Kang, Tae-Hyun Yoo

**Affiliations:** 1 Department of Internal Medicine, College of Medicine, Institute of Kidney Disease Research, Yonsei University, Seoul, Korea; 2 Department of Internal Medicine, College of Medicine, Severance Biomedical Science Institute, Brain Korea 21 PLUS, Yonsei University, Seoul, Korea; Hospital Universitario de la Princesa, SPAIN

## Abstract

**Background:**

Depression is reported to be the most common psychological problem in patients with chronic kidney disease (CKD). Several studies have reported that lower levels of serum vitamin D are significantly associated with depression. Both vitamin D deficiency and depression are prevalent in patients with CKD, yet the relationship between these two factors remains poorly understood. This study aimed to investigate the association between vitamin D levels and depression among CKD patients.

**Methods:**

Totally, 21,257 individuals who participated in the Korean National Health and Nutrition Examination Survey (KNHANES V, VI) from 2010–2014 were screened for the study; 533 CKD patients were included. Vitamin D deficiency was defined as serum 25-hydroxyvitamin D3 [25(OH)D3] ≤10 ng/mL. Patients were divided into vitamin D deficient or sufficient groups. Depression was screened for using the Korean version of the WHO Composite International Diagnostic Interview-Short Form. The association between vitamin D deficiency and depression was evaluated by multivariate logistic regression analysis.

**Results:**

The mean participant age was 70.1±9.4 years; 262 patients (49.2%) were male. The median 25(OH)D_3_ level was 19.1±6.9 ng/mL. The prevalence of depression was higher in CKD patients than in the general population (14.3 vs. 11.1%, P = 0.03). Additionally, the prevalence of depression was significantly higher in CKD patients with (vs. without) vitamin D deficiency (32.5% vs. 50.0%, P<0.001). Multivariate logistic regression analysis showed that vitamin D deficiency was a significant independent predictor of depression after adjusting for confounding factors (adjusted odds ratio, 6.15; 95% confidence interval, 2.02–8.75; P = 0.001).

**Conclusion:**

Depression was highly prevalent in CKD patients, in whom vitamin D deficiency was a significant independent predictor of depression. Therefore, management of vitamin D deficiency might help prevent depression in CKD patients.

## Introduction

Depression is reported to be the most common psychological problem in patients with chronic kidney disease (CKD) [1,2]. The prevalence of depression in patients with CKD is approximately 20–30%, which is higher than that of other chronic diseases [3,4]. Depression may result in poor quality of life and functional impairment, and may also hinder adherence to medical treatment and impair nutritional status [5,6]. Accumulating evidence has shown that depressive symptoms in patients undergoing dialysis are independent predictors of adverse clinical outcomes. The Dialysis Outcomes and Practice Patterns Study (DOPPS) showed a significant association between both mortality and hospitalization rates and depressive symptoms [7]. Moreover, high levels of depressive symptoms are found to be correlated with increased risk of cardiovascular events in an analysis from the Choices for Healthy Outcomes in Caring for End-Stage Renal Disease (CHOICE) study [8]. Recently, prospective studies found that patients with pre-dialysis CKD who experienced episodes of major depression were more likely to progress to dialysis [9,10].

Serum 25-hydroxyvitamin D_3_ [25(OH)D_3_] levels begin to decrease in stage 2 CKD [11,12], and deficiency of serum 25(OH)D_3_ is present in all subsequent stages of CKD [13–17], including end-stage renal disease (ESRD). Proteinuria may be accompanied by high urinary loss of vitamin D-binding protein, leading to increased renal loss of vitamin D metabolites [14,18,19]. Low serum 25(OH)D_3_ levels in patients with CKD have been associated with a higher risk of all-cause mortality and faster progression of kidney disease [20–22]. In the Third National Health and Nutrition Examination Survey (NHANES III) cohort [13], individuals with 25(OH)D_3_ levels lower than 15 ng/mL had a higher risk for all-cause mortality despite adjustments for CKD stage and confounding factors. Such findings have led to investigate on the role of 25(OH)D_3_ in multiple chronic diseases [23,24], and increase in serologic testing and use of vitamin D supplementation [25,26]. Various population and clinical research has implicated vitamin D deficiency as a potential risk factor for several chronic diseases, including hypertension, obesity, diabetes, cardiovascular diseases, autoimmune diseases, as well as depression [27–33]. Therefore, adequate vitamin D levels are a therapeutic goal for patients with kidney disease, in order to prevent several complications [34].

Several studies have reported that lower levels of serum 25(OH)D_3_ are significantly associated with depression [35–38]. Schneider et al. [39] suggested that psychiatric disorders, especially depression, may be associated with low levels of serum 25(OH)D_3_. Underlying causes of vitamin D deficiency, such as less sun exposure, can result from decreased outdoor physical activity, different housing or clothing habits, and decreased vitamin supplementation [40]. However, depression may also be a consequence of vitamin D deficiency. Serum 25(OH)D_3_ deficiency, while common, is highly treatable, which may help prevent depression.

As mentioned above, recent studies have reported the association between vitamin D deficiency and depression in the general population. Even though both vitamin D deficiency and depression are prevalent in patients with CKD, the relationship between these two factors remains poorly elucidated. Therefore, this study aimed to investigate the association between vitamin D levels and depression among patients with CKD.

## Methods

### Study design, setting, and participants

**Fig 1:**
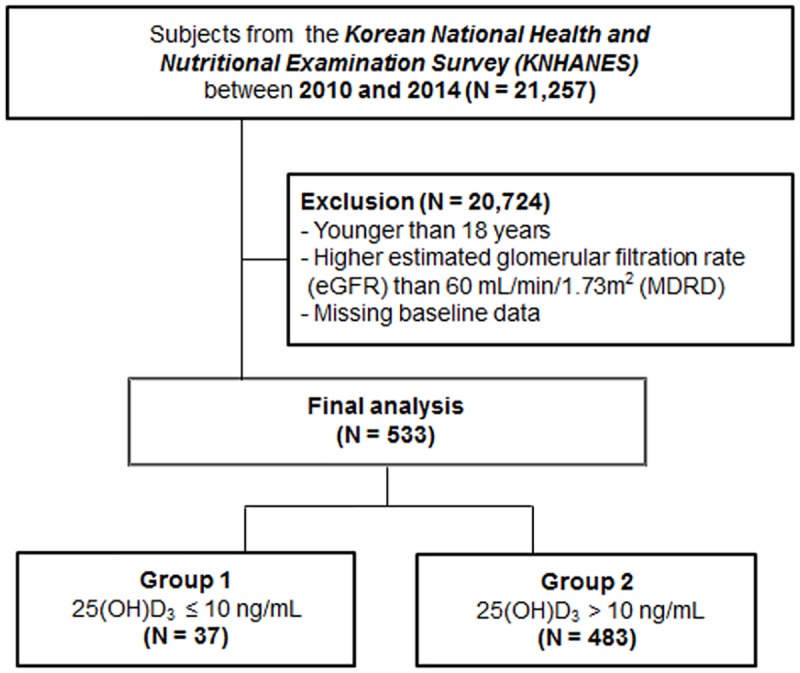
Flow diagram of study participant selection.

This study was based on data from the fifth and sixth editions of Korean National Health and Nutrition Examination Survey (KNHANES V, VI, 2010–2014, S1 Data). The KNHANES is a cross-sectional survey conducted periodically to assess the Korean public health and nutritional status. The KNHANES is composed of demographic, anthropometric, nutritional, and personal medical history data collected by trained investigators. Sampling frame was developed based on the multistage probability sampling that was stratified according to geographic location, gender, and age. In the fifth and sixth KNHANES (2010–2014), a total of 41,102 individuals aged ≥ 1 year were sampled. Among them, 21,257 individuals were screened for this study. Of the screened individuals, we only performed analyses of adults aged ≥ 19 years (Fig 1). We excluded individuals whose data were missing for important analytic variables, such as serum 25(OH)D_3_ levels, serum creatinine, and the mental health questionnaire. CKD was defined as an estimated glomerular filtration rate (eGFR) lower than 60 mL/min/1.73 m^2^ with or without proteinuria. Finally, 533 individuals who met the definition of CKD were included in the statistical analysis. The KNHANES was approved by the Institutional Review Board of the Centers for Disease Control and Prevention in Korea. All participants of the KNHANES used in this study provided their written informed consent.

### General characteristics of study participants

Anthropometric measurements were acquired by trained investigators following standardized protocols. Body mass index (BMI) was calculated as weight (kg)/squared height (m^2^). Demographic variables that were expected to be confounding factors included age, sex, marital status, occupational status, education status, economic status, alcohol behavior, smoking status, and EuroQol five dimensions (EQ5D) index score. Marital status was divided into two groups (single or married) based on questionnaire responses. Occupational status was divided into two groups: employed or unemployed. Education level was divided into three groups: elementary school, middle–high school, or college. Economic status was divided into three groups according to monthly income: low, middle, or high. The EQ5D descriptive system had five dimensions: mobility, self-care, usual activities, pain/discomfort, and anxiety/depression. A multi-attribute value function was used to map preferences for each of these health states. From this scoring algorithm, EQ5D index scores were calculated based on subjects’ responses to the five-item questionnaire. Scoring algorithm for the EQ5D index used in this study was based on the Korean value set [41].

#### Mental health

Mental health data were obtained by means of a self-reported mental health questionnaire, under the supervision of a trained investigator. Depressive symptoms were assessed by the following question: “In the past year, have you felt extreme sorrow or despair for more than two weeks?” Participants answered either “yes” or “no” to this question. This questionnaire had been used to identify people with depressive mood from the outset of KNHANES (1998) [42]. It is still widely used to measure participants with either clinical or subclinical depressive symptoms, which are expected to be substitutes for depressive disorders. In addition, participants were asked whether they had been diagnosed with depression by a physician. If subjects answered that they were either experiencing depressive symptoms or were currently diagnosed with depression, they were defined as having depression. Finally, participants were divided into two groups according to their answer, ‘‘depressive” or ‘‘non-depressive” group.

#### Serum 25(OH)D_3_ assessment and laboratory data

Blood samples, collected from the antecubital vein after a 10 to 12 hour fast, were used to assess serum levels of biochemical markers. Serum levels of 25(OH)D_3_ were measured by gamma counter with a radioimmunoassay (25-hydroxy-vitamin D125 I RIA Kit; DiaSorin, Stillwater, MN, USA) using a 1470 Wizard Gamma Counter (PerkinElmer, Turku, Finland). To make an effort for minimizing the analytical variation, serum 25(OH)D_3_ levels were analyzed by the same institute, which carried out a quality assurance program throughout the analysis period. Inter-assay coefficients of variation were 1.9–6.1% for the samples [43]. Participants were divided into two groups according to serum 25(OH)D_3_ levels: 25(OH)D_3_ deficient group (≤ 10 ng/mL) and 25(OH)D_3_ sufficient group (>10 ng/mL) [44]. In addition, biochemical laboratory test results for the following factors were collected: serum hemoglobin, glucose, total cholesterol, and glycosylated hemoglobin (Hba1c). Proteinuria was determined semi-quantitatively with a single spot urine dipstick analysis and reported as negative, trace, 1+, 2+, 3+, or 4+. Proteinuria was defined as 1+ or higher. The four-variable Modification of Diet in Renal Disease Study equation was used to calculate the eGFR [45].

#### Statistical analysis

All statistical analyses were performed using SPSS for Windows version 21.0 (SPSS Inc., Chicago, USA). Continuous variables were expressed as the mean ± standard deviation, and categorical variables as absolute number with percentages. Each variable was tested for normality before statistical analysis. Comparisons between the groups were made by the type of analysis for variance or Student’s t-test for continuous variables and by the chi-squared test or Fisher’s exact test for categorical variables. The Kolmogorov–Smirnov test was performed to determine the normality of the distribution of parameters. If the resulting data did not show a normal distribution, geometric mean ± standard deviation was reported; Either Mann–Whitney U test or Kruskal-Wallis test was used for multiple comparisons. Logistic regression analysis was done to evaluate the association between depressive symptom and serum 25(OH)D_3_ concentration. Odds ratios (ORs) for depressive symptoms were compared between 25(OH)D_3_ deficiency group and 25(OH)D_3_ sufficiency group. Multiple models were constructed for logistic regression. One was analyzed without adjustment, and others were adjusted for confounding factors including age, sex, smoking status, alcohol status, BMI, suicidal ideation, EQ5D index, past history of hypertension, diabetes mellitus, eGFR, proteinuria, serum glucose, hemoglobin, and total cholesterol level. P values less than 0.05 were considered statistically significant.

## Results

### Baseline characteristics of CKD patients according to 25(OH)D_3_ level

The baseline characteristics of study participants are shown in Table 1. The mean age was 70.1 ± 9.4 years, and 262 (49.2%) patients were male. The mean 25(OH)D_3_ level was 19.1 ± 6.9 ng/mL overall, 8.5 ± 1.3 ng/mL in the vitamin D deficient group, and 19.9 ± 6.5 ng/mL in the sufficient group. The vitamin D deficient group had a greater proportion of female and unemployed patients, and the EQ5D index that represents quality of life was lower. A past history of diabetes was prevalent in the vitamin D deficient group and the serum hemoglobin level was lower in the vitamin D deficient group. However, there were no differences in proteinuria, eGFR, or BMI between the two groups.

**Table 1:** Baseline characteristics of CKD patients.

	Total (n = 533)	Vit D Def (n = 27)	Vit D Suf (n = 483)	P
**Age (years)**	70.1 ± 9.4	71.6 ± 10.3	69.9 ± 9.4	0.30
**Male (%)**	262 (49.2)	13 (32.5)	249 (50.5)	0.03
**BMI (kg/m**^**2**^**)**	24.6 ± 3.4	24.2 ± 4.8	24.7 ± 3.3	0.36
**Smoker (%)**	256 (48.0)	14 (35.0)	242 (49.1)	0.09
**Alcohol user (%)**	260 (65.8)	15 (62.5)	245 (66.0)	0.73
**Mental health status:**				
**Suicidal ideation (%)**	104 (19.5)	8 (20.0)	96 (19.5)	0.9
**Depressive symptom (%)**	180 (33.8)	20 (50.0)	160 (32.5)	0.02
**EQ5D index**	0.84 ± 0.19	0.79 ± 0.21	0.85 ± 0.01	0.10
**Stress (%):**				0.23
None	151 (28.9)	14 (36.8)	137 (28.3)	
Mild	276 (52.9)	15 (39.5)	261 (53.9)	
Moderate to severe	95 (18.2)	9 (23.7)	86 (17.8)	
**Sleeping hours (%)**				0.07
< 6 hours	31 (6.1)	5 (12.8)	26 (5.5)	
6–9 hours	334 (65.7)	20 (51.3)	314 (67.0)	
>10 hours	143 (28.1)	14 (35.9)	129 (27.5)	
**Socioeconomic status (%):**				
**Married**	527 (99.1)	39 (97.5)	488 (99.2)	
**Economic status**				0.23
Low	129 (24.6)	11 (28.9)	118 (24.2)	
Middle	258 (49.1)	19 (50.0)	239 (49.1)	
High	138 (26.3)	8 (21.1)	130 (26.7)	
**Education**				0.9
≤ Elementary	292 (56.2)	22 (57.9)	270 (56.0)	
Middle-high school	187 (36.0)	13 (34.2)	174 (36.1)	
≥ College	41 (7.9)	3 (7.9)	38 (7.9)	
**Employed**	169 (32.6)	4 (10.5)	165 (34.3)	0.003
**Comorbidities (%):**				
HTN	379 (71.1)	27 (67.5)	352 (71.4)	0.60
DM	168 (31.5)	22 (55.0)	146 (29.6)	0.001
CVD	102 (19.1)	7 (17.5)	95 (19.3)	0.78
Dyslipidemia	145 (27.2)	12 (30.0)	133 (27.0)	0.68
**Laboratory data:**				
Hemoglobin (g/dl)	13.2 ± 1.8	12.3 ± 1.6	13.2 ± 1.8	0.002
eGFR (ml/min/1.73 m^2^)	50.5 ± 9.8	48.7 ± 11.2	50.6 ± 9.7	0.24
Proteinuria (%)	52 (9.8)	5 (12.5)	47 (9.5)	0.54
Total cholesterol (mg/dl)	186.2 ± 41.3	176.6 ± 39.3	187.0 ± 41.4	0.13
Glucose (mg/dl)	108.9 ± 30.4	109.7 ± 30.1	108.8 ± 30.5	0.87
25(OH)D_3_ (ng/ml)	19.1 ± 6.9	8.5 ± 1.3	19.9 ± 6.5	<0.001

Data are presented as mean ± SD or n (%).

Abbreviations: Vit D Def, vitamin D deficiency; Vit D Suf, vitamin D sufficiency; CKD, chronic kidney disease; BMI, body mass index; HTN, hypertension; DM, diabetes mellitus; CVD, cardiovascular disease; eGFR, estimated glomerular filtration rate

### Comparison of prevalence of depression between the general population and CKD patients

**Fig 2:**
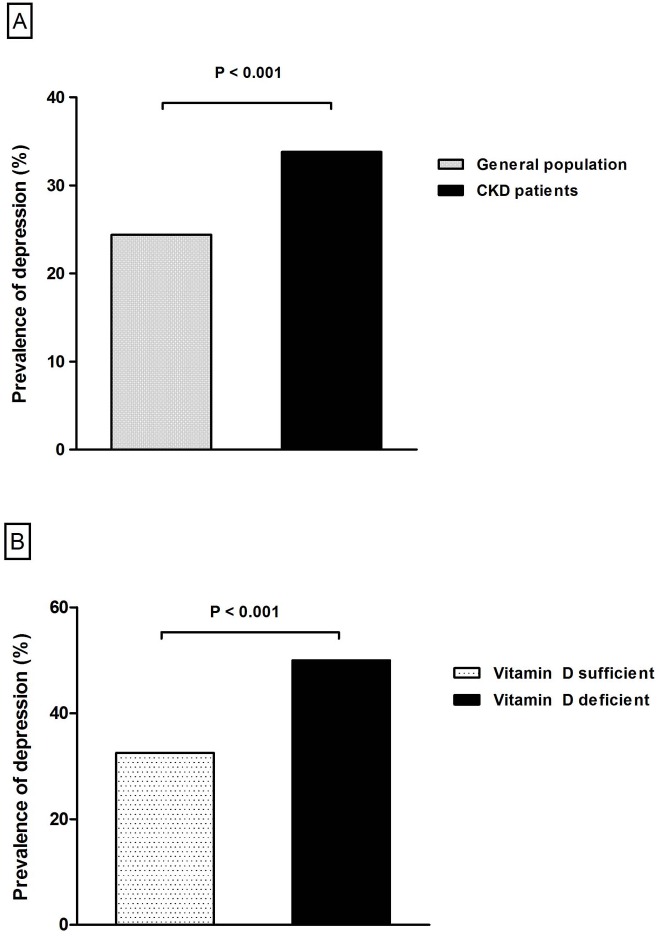
(A) Prevalence of depression between the general population and CKD patients (B) Prevalence of depression between vitamin D deficient and sufficient group among CKD patients.

Chi-square analysis was performed to compare the prevalence of depression between CKD patients and the general population (Fig 2A). The prevalence of depression was significantly higher in CKD patients compared with the general population (24.4% versus 33.8%, P < 0.001).

### Comparison of prevalence of depression between the vitamin D deficient and sufficient groups of CKD patients

We further evaluated the association of depression and vitamin D deficiency in CKD patients. The prevalence of depression was significantly higher in the vitamin D deficient group of CKD patients, compared with those with sufficient levels of vitamin D (32.5% versus 50.0%, P < 0.001, Fig 2B).

### Vitamin D deficiency as a predictor of depression in CKD patients

Logistic regression analysis was performed to investigate the independent association between vitamin D deficiency and depression (Table 2). In the crude logistic regression analysis, vitamin D deficiency was significantly associated with depression [OR, 2.08; 95% confidence interval (CI), 1.09–3.98; P = 0.03]. In the multivariate logistic regression analysis, the significant association remained, even after adjustment for factors reported to be associated with depression, such as age, sex, alcohol status, smoking status, suicidal ideation, occupational status, education status, economic status, EQ5D index, past history of hypertension or diabetes, serum hemoglobin level, eGFR, proteinuria, and body mass index (OR, 6.15; 95% CI, 2.02–18.75; P = 0.001).

**Table 2:** Logistic regression analysis for the prevalence of depression with adjustment for various factors.

	Crude^a^		Model 1^b^		Model 2^c^		Model 3^d^	
	OR (95% CI)	P	OR (95% CI)	P	OR (95% CI)	P	OR (95% CI)	P
**Vit. D deficiency**	2.08 (1.09–3.98)	0.03	5.13 (1.81–4.52)	0.002	5.02 (1.75–14.42)	0.003	6.15 (2.02–8.75)	0.001
**Age**			0.97 (0.93–1.01)	0.14	0.97 (0.93–5.75)	0.15	0.97 (0.93–1.01)	0.14
**Male**			1.68 (0.50–5.59)	0.40	1.65 (0.49–5.58)	0.42	1.35 (0.37–4.10)	0.65
**Smoking status**			1.71 (0.34–3.38)	0.9	1.05 (0.33–3.36)	0.9	1.01 (0.31–3.26)	0.9
**Alcohol behavior**			0.89 (0.43–1.82)	0.74	0.89 (0.43–1.84)	0.76	0.83 (0.39–1.74)	0.62
**Occupation**			1.06 (0.47–2.40)	0.89	1.07 (0.47–2.42)	0.88	1.08 (0.47–2.48)	0.85
**Economic status**			0.94 (0.56–1.59)	0.81	0.94 (0.56–1.59)	0.82	0.93 (0.55–1.58)	0.79
**Education status**			1.32 (0.71–2.46)	0.37	1.32 (0.71–2.45)	0.38	1.40 (0.74–2.64)	0.30
**Suicidal ideation**			NA	0.9	NA	0.9	NA	0.9
**EQ5D index**			0.94 (0.92–0.96)	<0.001	0.94 (0.92–0.96)	<0.001	0.94 (0.92–0.96)	<0.001
**HTN**					0.98 (0.46–2.06)	0.9	0.91 (0.42–2.01)	0.82
**DM**					1.09 (0.52–2.28)	0.81	1.00 (0.46–2.17)	0.9
**eGFR**							0.99 (0.96–1.03)	0.71
**Proteinuria**							0.45 (0.13–1.63)	0.23
**Hemoglobin**							0.94 (0.73–1.20)	0.56
**BMI**							1.08 (0.96–1.21)	0.20

^a^ Unadjusted model

^b^ Adjusted for age, sex, alcohol, smoking, suicidal idea, and EQ5D index

^c^ Adjusted for Model 1 + HTN and DM

^d^ Adjusted for Model 2 + hemoglobin, glucose, total cholesterol, eGFR, proteinuria, and BMI

Abbreviations: OR, odds ratio; CI, confidence interval; Vit. D, vitamin D; NA, not applicable; HTN, hypertension; DM, diabetes mellitus; eGFR, estimated glomerular filtration rate; BMI, body mass index

### Subgroup analysis

Subgroup analysis was conducted according to age, sex, EQ5D index, and presence of diabetes (Table 3). In the group aged < 65 years, vitamin D deficiency and depression were significantly associated after adjustment for confounding factors (OR = 398.57; 95% CI: 10.68–14867.71; P = 0.001). After adjusting for confounding factors, vitamin D deficiency and depression were significantly associated in female (OR, 42.89; 95% CI, 5.22–352.35; P < 0.001) but not in male (OR, 0.58; 95% CI, 0.05–6.25; P = 0.65). The patients were divided into two groups according to EQ5D index. In the higher EQ5D index group, the association between vitamin D deficiency and depression was significant, even after adjustment for confounding variables (OR, 7.49; 95% CI, 1.41–39.74; P = 0.02), whereas in lower EQ5D index group, there was no significant association (OR, 3.91; 95% CI, 0.68–22.63; P = 0.13). Patients with no history of diabetes showed similar results to those above (OR, 18.94; 95% CI, 3.41–105.03; P = 0.001).

**Table 3:** Subgroup analysis according to age, sex, EQ5D index, and presence of diabetes with multiple logistic regression analysis for the presence of depression.

Subgroup	Variable	OR (95% CI)	P
**Age**			
< 65	Vitamin D deficiency	398.57 (10.68–14867.71)	0.001
≥ 65	Vitamin D deficiency	2.23 (0.49–10.01)	0.30
**Sex**			
Female	Vitamin D deficiency	42.88 (5.22–352.35)	<0.001
Male	Vitamin D deficiency	0.58 (0.05–6.25)	0.65
**EQ5D index** ^a^			
High	Vitamin D deficiency	7.49 (1.41–39.74)	0.02
Low	Vitamin D deficiency	3.91 (0.67–22.63)	0.13
**Diabetes mellitus**			
No	Vitamin D deficiency	18.94 (3.41–105.03)	0.001
Yes	Vitamin D deficiency	5.00 (0.76–32.84)	0.09

^a^ Subgroup was divided according to EQ5D index. The mean values of EQ5D index were 0.98 and 0.71 in the high and low groups, respectively.

## Discussion

This study showed that vitamin D deficiency was significantly associated with depressive symptoms in patients with CKD. Consistent with previous reports, the prevalence of depression was significantly higher in these individuals compared with the general population. Among patients with CKD, depression was more prevalent in the vitamin D deficiency group. Furthermore, vitamin D deficiency was an independent predictor of depression in patients with CKD, even after adjustment for confounders. To the best of our knowledge, this is the first investigation elucidating a relationship between vitamin D deficiency and depressive symptoms in patients with CKD.

In this study, the prevalence of depression in CKD subjects was significantly higher compared with the general population. Furthermore, this study showed that vitamin D deficiency was an independent predictor for the presence of depression even after adjustment for multiple confounding factors in CKD patients. Although the cause of depression in patients with CKD is multifactorial, vitamin D deficiency is one of the plausible factors that has recently garnered much interest [46]. Vitamin D is a distinctive neurosteroid hormone and may play an important role in the development of depression. Vitamin D receptors are present extensively on neurons and glia in many areas of the brain, including the cingulate cortex and hippocampus, which have been implicated in the pathophysiology of depression [47]. Vitamin D also promotes the synthesis of depression-related monoamine neurotransmitters, such as serotonin, and is involved in numerous brain processes, including neuroimmuno-modulation, regulation of neurotrophic factors, neuroprotection, neuroplasticity, and brain development, making it biologically reasonable that vitamin D deficiency might contribute to the development of depression [48,49]. In addition, regarding physical functioning, vitamin D receptors have been observed in the cerebellum, which is an important area of the brain for mobility, gait, and balance [47], and in muscle tissue, which facilitates muscle contraction speed, muscle power, and cell growth [48]. Vitamin D deficiency might attenuate muscle power and further lead to poor physical functioning. It is well known that reduced physical activity is a risk factor for depression [50]. For reasons mentioned above, vitamin D deficiency can lead to depression.

Several risk factors for vitamin D deficiency have been identified, some of which are also associated with depression. These include aging, female sex, dark skin pigmentation, winter season, obesity, impaired liver function, no use of vitamin D supplements, antidepressant and anticonvulsant medication use, and psychiatric comorbidities such as anxiety or eating disorders [51]. Furthermore, a range of medical conditions, including cardiovascular, liver, and renal disease may be associated with low vitamin D levels [52]. However, to date, even though both vitamin D deficiency and depression are prevalent in patients with CKD, the relationship between these two factors in this group of patients has been poorly elucidated. In this regard, this study is notable in that it is the first to investigate the association between vitamin D deficiency and depression in CKD patients. In this study, CKD patients with low vitamin D levels were older, more likely to have diabetes, and had lower levels of hemoglobin. These factors are known to be markers of poor medical condition. This poor state of health can be one of the risk factors for depressive symptoms. Thus, poor medical condition in the group with vitamin D deficiency could be a reason for the relationship between vitamin D deficiency and depression.

It is well known that the blood level of vitamin D in patients with CKD is low [53]. Limited exposure to sunlight in combination with impaired UVB-induced vitamin D synthesis in the skin and disturbed vitamin D metabolism are considered to contribute to low 25(OH)D_3_ levels in patients with CKD [54,55]. As previously mentioned, vitamin D deficiency is a plausible risk factor for depressive symptoms. According to the results of the present work, the mean serum 25(OH)D_3_ level was 19.1 ng/mL in CKD patients. Hence, almost all CKD patients in this study experienced vitamin D insufficiency. Thus, this study showed that in patients with CKD, the prevalence of depressive symptoms is high and, given that vitamin D deficiency in these patients is highly prevalent [53], management of vitamin D deficiency in CKD is beneficial to prevent the development of depression. A randomized double blind trial reported that supplementation of vitamin D ameliorates depressive symptoms [56]. This result indicates that vitamin D deficiency is associated with depressive symptoms. However, to date, there is no randomized control trial targeting CKD patients investigating the relationship between vitamin D supplementation and depression. Further studies are needed to support the association of vitamin D deficiency and depression. Specifically, subgroup analysis revealed that the strength of associations still had significance in those aged <65 years, in women, in those with no history of diabetes, and the group whose EQ5D index was high. However, among those aged ≥ 65, with diabetes, or with a low EQ5D index, no association with vitamin D deficiency and depression was demonstrated. This is likely because patients who are older, have a poor medical condition, or have a low EQ5D index have a more complicated condition so that other confounding factors influence the development of depression. However, our results could be generalizable to CKD patients who are young, female, and have less complicated medical disease. Among these patients, screening for vitamin D deficiency might prevent depression.

This study has several limitations. First, as serum vitamin D level is generally affected by the extent of sun exposure, seasonal variation should be considered. However, because the data used in this study could not be linked to specific dates of laboratory measure, seasonal variation could not be included. Second, since the liver is a key organ involved in the metabolism of vitamin D, a history of liver disease may affect vitamin D deficiency. However, in this study, only a few subjects had liver disease; hence, we did not adjust for this. Third, serum PTH level is associated with vitamin D deficiency and depression. Furthermore, it is well-known that active vitamin D metabolite is more significant in CKD patients. However, since KNHANES focused on the basic public nutritional status, it does not provide special laboratory findings such as PTH level and active vitamin D metabolite. Finally, there is paucity of data from randomized control studies on whether supplementation of vitamin D can improve or prevent depression or adverse clinical outcomes. Further randomized control trials should be performed to determine whether vitamin D supplementation could be beneficial to improve depression and adverse clinical outcomes in patients with CKD as well as in the general population.

In conclusion, it is notable that this study demonstrated the association between vitamin D deficiency and depression in CKD patients. Further randomized control trials should be performed to determine whether vitamin D supplementation could be beneficial to improve depression and adverse clinical outcomes in CKD patients as well as in the general population.

## Supporting information

S1 Data Data set files used for the study analysis. (SAV)

## References

[1] Hedayati SS, Minhajuddin AT, Toto RD, Morris DW, Rush AJ. Prevalence of major depressive episode in CKD. Am J Kidney Dis. 2009;54: 424–432. 10.1053/j.ajkd.2009.03.017 19493599 PMC3210064

[2] Tsai YC, Chiu YW, Hung CC, Hwang SJ, Tsai JC, Wang SL, et al Association of symptoms of depression with progression of CKD. Am J Kidney Dis. 2012;60: 54–61. 10.1053/j.ajkd.2012.02.325 22495469

[3] Murtagh FE, Addington-Hall J, Higginson IJ. The prevalence of symptoms in end-stage renal disease: a systematic review. Adv Chronic Kidney Dis. 2007;14: 82–99. 10.1053/j.ackd.2006.10.001 17200048

[4] Chan R, Steel Z, Brooks R, Heung T, Erlich J, Chow J, et al Psychosocial risk and protective factors for depression in the dialysis population: a systematic review and meta-regression analysis. J Psychosom Res. 2011;71: 300–310. 10.1016/j.jpsychores.2011.05.002 21999973

[5] Hedayati SS, Bosworth HB, Kuchibhatla M, Kimmel PL, Szczech LA. The predictive value of self-report scales compared with physician diagnosis of depression in hemodialysis patients. Kidney Int. 2006;69: 1662–1668. 10.1038/sj.ki.5000308 16598203

[6] Cukor D, Rosenthal DS, Jindal RM, Brown CD, Kimmel PL. Depression is an important contributor to low medication adherence in hemodialyzed patients and transplant recipients. Kidney Int. 2009;75: 1223–1229. 10.1038/ki.2009.51 19242502

[7] Lopes AA, Bragg J, Young E, Goodkin D, Mapes D, Combe C, et al Depression as a predictor of mortality and hospitalization among hemodialysis patients in the United States and Europe. Kidney Int. 2002;62: 199–207. 10.1046/j.1523-1755.2002.00411.x 12081579

[8] Boulware LE, Liu Y, Fink NE, Coresh J, Ford DE, Klag MJ, et al Temporal relation among depression symptoms, cardiovascular disease events, and mortality in end-stage renal disease: contribution of reverse causality. Clin J Am Soc Nephrol. 2006;1: 496–504. 10.2215/CJN.00030505 17699251

[9] Hedayati SS, Minhajuddin AT, Afshar M, Toto RD, Trivedi MH, Rush AJ. Association between major depressive episodes in patients with chronic kidney disease and initiation of dialysis, hospitalization, or death. JAMA. 2010;303: 1946–1953. 10.1001/jama.2010.619 20483971 PMC3217259

[10] Palmer SC, Vecchio M, Craig JC, Tonelli M, Johnson DW, Nicolucci A, et al Association between depression and death in people with CKD: a meta-analysis of cohort studies. Am J Kidney Dis. 2013;62: 493–505. 10.1053/j.ajkd.2013.02.369 23623139

[11] Rickers H, Christiansen C, Christensen P, Christensen M, Rodbro P. Serum concentrations of vitamin D metabolites in different degrees of impaired renal function. Estimation of renal and extrarenal secretion rate of 24,25-dihydroxyvitamin D. Nephron. 1985;39: 267–271. 3871920 10.1159/000183383

[12] Reichel H, Deibert B, Schmidt-Gayk H, Ritz E. Calcium metabolism in early chronic renal failure: implications for the pathogenesis of hyperparathyroidism. Nephrol Dial Transplant. 1991;6: 162–169. 1866044 10.1093/ndt/6.3.162

[13] Mehrotra R, Kermah DA, Salusky IB, Wolf MS, Thadhani RI, Chiu YW, et al Chronic kidney disease, hypovitaminosis D, and mortality in the United States. Kidney Int. 2009;76: 977–983. 10.1038/ki.2009.288 19657329 PMC3791220

[14] Gonzalez EA, Sachdeva A, Oliver DA, Martin KJ. Vitamin D insufficiency and deficiency in chronic kidney disease. A single center observational study. Am J Nephrol. 2004;24: 503–510. 10.1159/000081023 15452403

[15] LaClair RE, Hellman RN, Karp SL, Kraus M, Ofner S, Li Q, et al Prevalence of calcidiol deficiency in CKD: a cross-sectional study across latitudes in the United States. Am J Kidney Dis. 2005;45: 1026–1033. 15957131 10.1053/j.ajkd.2005.02.029

[16] Levin A, Bakris GL, Molitch M, Smulders M, Tian J, Williams LA, et al Prevalence of abnormal serum vitamin D, PTH, calcium, and phosphorus in patients with chronic kidney disease: results of the study to evaluate early kidney disease. Kidney Int. 2007;71: 31–38. 10.1038/sj.ki.5002009 17091124

[17] Pilz S, Iodice S, Zittermann A, Grant WB, Gandini S. Vitamin D status and mortality risk in CKD: a meta-analysis of prospective studies. Am J Kidney Dis. 2011;58: 374–382. 10.1053/j.ajkd.2011.03.020 21636193

[18] Koenig KG, Lindberg JS, Zerwekh JE, Padalino PK, Cushner HM, Copley JB. Free and total 1,25-dihydroxyvitamin D levels in subjects with renal disease. Kidney Int. 1992;41: 161–165. 1593853 10.1038/ki.1992.22

[19] Sato KA, Gray RW, Lemann J Jr. Urinary excretion of 25-hydroxyvitamin D in health and the nephrotic syndrome. J Lab Clin Med. 1982;99: 325–330. 6977006

[20] Drechsler C, Pilz S, Obermayer-Pietsch B, Verduijn M, Tomaschitz A, Krane V, et al Vitamin D deficiency is associated with sudden cardiac death, combined cardiovascular events, and mortality in haemodialysis patients. Eur Heart J. 2010;31: 2253–2261. 10.1093/eurheartj/ehq246 20688781 PMC2938469

[21] Drechsler C, Verduijn M, Pilz S, Dekker FW, Krediet RT, Ritz E, et al Vitamin D status and clinical outcomes in incident dialysis patients: results from the NECOSAD study. Nephrol Dial Transplant. 2011;26: 1024–1032. 10.1093/ndt/gfq606 20947538

[22] Ravani P, Malberti F, Tripepi G, Pecchini P, Cutrupi S, Pizzini P, et al Vitamin D levels and patient outcome in chronic kidney disease. Kidney Int. 2009;75: 88–95. 10.1038/ki.2008.501 18843258

[23] Pilz S, Dobnig H, Winklhofer-Roob B, Riedmuller G, Fischer JE, Seelhorst U, et al Low serum levels of 25-hydroxyvitamin D predict fatal cancer in patients referred to coronary angiography. Cancer Epidemiol Biomarkers Prev. 2008;17: 1228–1233. 10.1158/1055-9965.EPI-08-0002 18463400

[24] Tuohimaa P, Tenkanen L, Ahonen M, Lumme S, Jellum E, Hallmans G, et al Both high and low levels of blood vitamin D are associated with a higher prostate cancer risk: a longitudinal, nested case-control study in the Nordic countries. Int J Cancer. 2004;108: 104–108. 10.1002/ijc.11375 14618623

[25] Gahche J, Bailey R, Burt V, Hughes J, Yetley E, Dwyer J, et al Dietary supplement use among U.S. adults has increased since NHANES III (1988–1994). NCHS Data Brief. 2011 2011/05/20: 1–8. 21592424

[26] Hollis BW. Measuring 25-hydroxyvitamin D in a clinical environment: challenges and needs. Am J Clin Nutr. 2008;88: 507s–510s. 18689391 10.1093/ajcn/88.2.507S

[27] Zittermann A, Iodice S, Pilz S, Grant WB, Bagnardi V, Gandini S. Vitamin D deficiency and mortality risk in the general population: a meta-analysis of prospective cohort studies. Am J Clin Nutr. 2012;95: 91–100. 10.3945/ajcn.111.014779 22170374

[28] Bolland MJ, Grey A, Gamble GD, Reid IR. Calcium and vitamin D supplements and health outcomes: a reanalysis of the Women's Health Initiative (WHI) limited-access data set. Am J Clin Nutr. 2011;94: 1144–1149. 10.3945/ajcn.111.015032 21880848 PMC3173029

[29] Formiga F, Ferrer A, Fraga A, Pujol R. [Vitamin D levels and mortality of any cause in nonagenarians. NonaSantfeliu Study]. Med Clin (Barc). 2011;137: 137–138. 20207376 10.1016/j.medcli.2010.01.014

[30] Cawthon PM, Parimi N, Barrett-Connor E, Laughlin GA, Ensrud KE, Hoffman AR, et al Serum 25-hydroxyvitamin D, parathyroid hormone, and mortality in older men. J Clin Endocrinol Metab. 2010;95: 4625–4634. 10.1210/jc.2010-0638 20631024 PMC3050100

[31] Grant WB, Schwalfenberg GK, Genuis SJ, Whiting SJ. An estimate of the economic burden and premature deaths due to vitamin D deficiency in Canada. Mol Nutr Food Res. 2010;54: 1172–1181. 10.1002/mnfr.200900420 20352622

[32] de Boer IH, Kestenbaum B, Shoben AB, Michos ED, Sarnak MJ, Siscovick DS. 25-hydroxyvitamin D levels inversely associate with risk for developing coronary artery calcification. J Am Soc Nephrol. 2009;20: 1805–1812. 10.1681/ASN.2008111157 19443637 PMC2723983

[33] Jorde R, Figenschau Y. Supplementation with cholecalciferol does not improve glycaemic control in diabetic subjects with normal serum 25-hydroxyvitamin D levels. Eur J Nutr. 2009;48: 349–354. 10.1007/s00394-009-0020-3 19370371

[34] KDOQI Clinical Practice Guideline for Diabetes and CKD: 2012 Update. Am J Kidney Dis. 2012;60: 850–886. 10.1053/j.ajkd.2012.07.005 23067652

[35] Wilkins CH, Sheline YI, Roe CM, Birge SJ, Morris JC. Vitamin D deficiency is associated with low mood and worse cognitive performance in older adults. Am J Geriatr Psychiatry. 2006;14: 1032–1040. 10.1097/01.JGP.0000240986.74642.7c 17138809

[36] Jorde R, Waterloo K, Saleh F, Haug E, Svartberg J. Neuropsychological function in relation to serum parathyroid hormone and serum 25-hydroxyvitamin D levels. The Tromso study. J Neurol. 2006;253: 464–470. 10.1007/s00415-005-0027-5 16283099

[37] Eskandari F, Martinez PE, Torvik S, Phillips TM, Sternberg EM, Mistry S, et al Low bone mass in premenopausal women with depression. Arch Intern Med. 2007;167: 2329–2336. 10.1001/archinte.167.21.2329 18039992

[38] Hoogendijk WJ, Lips P, Dik MG, Deeg DJ, Beekman AT, Penninx BW. Depression is associated with decreased 25-hydroxyvitamin D and increased parathyroid hormone levels in older adults. Arch Gen Psychiatry. 2008;65: 508–512. 10.1001/archpsyc.65.5.508 18458202

[39] Schneider B, Weber B, Frensch A, Stein J, Fritz J. Vitamin D in schizophrenia, major depression and alcoholism. J Neural Transm (Vienna). 2000;107: 839–842. 11005548 10.1007/s007020070063

[40] Michelson D, Stratakis C, Hill L, Reynolds J, Galliven E, Chrousos G, et al Bone mineral density in women with depression. N Engl J Med. 1996;335: 1176–1181. 10.1056/NEJM199610173351602 8815939

[41] Lee YK, Nam HS, Chuang LH, Kim KY, Yang HK, Kwon IS, et al South Korean time trade-off values for EQ-5D health states: modeling with observed values for 101 health states. Value Health. 2009;12: 1187–1193. 10.1111/j.1524-4733.2009.00579.x 19659703

[42] Park SJ, Jeon HJ, Kim JY, Kim S, Roh S. Sociodemographic factors associated with the use of mental health services in depressed adults: results from the Korea National Health and Nutrition Examination Survey (KNHANES). BMC Health Serv Res. 2014;14: 645 10.1186/s12913-014-0645-7 25527283 PMC4307909

[43] Chung HK, Cho Y, Choi S, Shin MJ. The association between serum 25-hydroxyvitamin D concentrations and depressive symptoms in Korean adults: findings from the fifth Korea National Health and Nutrition Examination Survey 2010. PLoS One. 2014;9: e99185 10.1371/journal.pone.0099185 24945632 PMC4063710

[44] Holick MF, Binkley NC, Bischoff-Ferrari HA, Gordon CM, Hanley DA, Heaney RP, et al Evaluation, treatment, and prevention of vitamin D deficiency: an Endocrine Society clinical practice guideline. J Clin Endocrinol Metab. 2011;96: 1911–1930. 10.1210/jc.2011-0385 21646368

[45] Levey AS, Bosch JP, Lewis JB, Greene T, Rogers N, Roth D. A more accurate method to estimate glomerular filtration rate from serum creatinine: a new prediction equation. Modification of Diet in Renal Disease Study Group. Ann Intern Med. 1999;130: 461–470. 10075613 10.7326/0003-4819-130-6-199903160-00002

[46] Anglin RE, Samaan Z, Walter SD, McDonald SD. Vitamin D deficiency and depression in adults: systematic review and meta-analysis. Br J Psychiatry. 2013;202: 100–107. 10.1192/bjp.bp.111.106666 23377209

[47] Eyles DW, Smith S, Kinobe R, Hewison M, McGrath JJ. Distribution of the vitamin D receptor and 1 alpha-hydroxylase in human brain. J Chem Neuroanat. 2005;29: 21–30. 10.1016/j.jchemneu.2004.08.006 15589699

[48] Fernandes de Abreu DA, Eyles D, Feron F. Vitamin D, a neuro-immunomodulator: implications for neurodegenerative and autoimmune diseases. Psychoneuroendocrinology. 2009;34 Suppl 1: S265–277. 19545951 10.1016/j.psyneuen.2009.05.023

[49] Kesby JP, Eyles DW, Burne TH, McGrath JJ. The effects of vitamin D on brain development and adult brain function. Mol Cell Endocrinol. 2011;347: 121–127. 10.1016/j.mce.2011.05.014 21664231

[50] Helgadottir B, Forsell Y, Ekblom O. Physical activity patterns of people affected by depressive and anxiety disorders as measured by accelerometers: a cross-sectional study. PLoS One. 2015;10: e0115894 10.1371/journal.pone.0115894 25585123 PMC4293141

[51] Maddock J, Berry DJ, Geoffroy MC, Power C, Hypponen E. Vitamin D and common mental disorders in mid-life: cross-sectional and prospective findings. Clin Nutr. 2013;32: 758–764. 10.1016/j.clnu.2013.01.006 23395104

[52] von Kanel R, Fardad N, Steurer N, Horak N, Hindermann E, Fischer F, et al Vitamin D Deficiency and Depressive Symptomatology in Psychiatric Patients Hospitalized with a Current Depressive Episode: A Factor Analytic Study. PLoS One. 2015;10: e0138550 10.1371/journal.pone.0138550 26397113 PMC4580407

[53] K/DOQI clinical practice guidelines for bone metabolism and disease in chronic kidney disease. Am J Kidney Dis. 2003;42: S1–201. 14520607

[54] Mehrotra R, Kermah D, Budoff M, Salusky IB, Mao SS, Gao YL, et al Hypovitaminosis D in chronic kidney disease. Clin J Am Soc Nephrol. 2008;3: 1144–1151. 10.2215/CJN.05781207 18417740 PMC2440286

[55] Jacob AI, Sallman A, Santiz Z, Hollis BW. Defective photoproduction of cholecalciferol in normal and uremic humans. J Nutr. 1984;114: 1313–1319. 6330321 10.1093/jn/114.7.1313

[56] Jorde R, Sneve M, Figenschau Y, Svartberg J, Waterloo K. Effects of vitamin D supplementation on symptoms of depression in overweight and obese subjects: randomized double blind trial. J Intern Med. 2008;264: 599–609. 10.1111/j.1365-2796.2008.02008.x 18793245

